# The effect of Cyclophilin D depletion on liver regeneration following associating liver partition and portal vein ligation for staged hepatectomy

**DOI:** 10.1371/journal.pone.0271606

**Published:** 2022-07-14

**Authors:** Noemi Daradics, Gergo Horvath, Laszlo Tretter, Agnes Paal, Andras Fulop, Andras Budai, Attila Szijarto

**Affiliations:** 1 Department of Surgery, Transplantation and Interventional Gastroenterology, Semmelweis University, Hepato-Pancreatico-Biliary (HPB) Surgical Research Center Hungary, Budapest, Hungary; 2 Department of Medical Biochemistry, Semmelweis University, Budapest, Hungary; 3 2^nd^ Department of Pathology, Semmelweis University, Budapest, Hungary; Montana State University Bozeman, UNITED STATES

## Abstract

**Aim:**

Associating Liver Partition and Portal vein ligation for Staged hepatectomy (ALPPS) is a modification of two-stage hepatectomy profitable for patients with inoperable hepatic tumors by standard techniques. Unfortunately, initially poor postoperative outcome was associated with ALPPS, in which mitochondrial dysfunction played an essential role. Inhibition of cyclophilins has been already proposed to be efficient as a mitochondrial therapy in liver diseases. To investigate the effect of Cyclophilin D (CypD) depletion on mitochondrial function, biogenesis and liver regeneration following ALPPS a CypD knockout (KO) mice model was created.

**Methods:**

Male wild type (WT) (n = 30) and CypD KO (n = 30) mice underwent ALPPS procedure. Animals were terminated pre-operatively and 24, 48, 72 or 168 h after the operation. Mitochondrial functional studies and proteomic analysis were performed. Regeneration rate and mitotic activity were assessed.

**Results:**

The CypD KO group displayed improved mitochondrial function, as both ATP production (P < 0.001) and oxygen consumption (P < 0.05) were increased compared to the WT group. The level of mitochondrial biogenesis coordinator peroxisome proliferator-activated receptor γ co-activator 1-α (PGC1-α) was also elevated in the CypD KO group (P < 0.001), which resulted in the induction of the mitochondrial oxidative phosphorylation system. Liver growth increased in the CypD KO group compared to the WT group (P < 0.001).

**Conclusions:**

Our study demonstrates the beneficial effect of CypD depletion on the mitochondrial vulnerability following ALPPS. Based on our results we propose that CypD inhibition should be further investigated as a possible mitochondrial therapy following ALPPS.

## 1. Introduction

For patients with liver cancer the main restricting factor of curative resection is currently the insufficient volume of future liver remnant (FLR) following surgery, which could lead to fatal outcome by inducing post-hepatectomy liver failure [1]. Associating Liver Partition and Portal vein ligation for Staged hepatectomy (ALPPS) is a modification of two-stage hepatectomy, which could enhance a robust and rapid liver growth, offering the possibility of curative therapy for patients that are considered inoperable by conventional two-stage hepatectomy. Apart from the remarkable advantages, initially high morbidity (up to 80.6 percent) and mortality rates (up to 28.7 percent) were associated with ALPPS [2]. Since then, due to better patient selection and technical modifications these rates decreased [3], however the underlying pathomechanism is still unclear.

Besides of the rapid increase of the liver volume, there may be a lagging functional recovery following ALPPS [4]. According to our previous studies [5,6], impaired mitochondrial function and biogenesis could explain the inadequate functional regeneration. Comparing ALPPS to conventional two-stage hepatectomy, we found that while mitochondrial function and mitochondrial biogenesis pathway coordinator peroxisome proliferator-activated receptor γ co-activator 1-α (PGC1-α) along with its transcription factor Nuclear Respiratory Factor (NRF) 1 were preserved after conventional two-stage hepatectomy, they decreased and remained impaired after ALPPS. This, along with the robust energy-demanding regeneration led to an energetic imbalance [5]. Proceeding with our studies on mitochondrial function after ALPPS, we demonstrated that preoperative exercise applied as physical prehabilitation could significantly enhance mitochondrial function and biogenesis along with an even more powerful regeneration [6].

According to the above, improving mitochondrial dysfunction could help achieve better outcomes following ALPPS. The inhibition of Cyclophilin D (CypD) has recently been introduced as a mitochondrial therapy in liver diseases [7]. CypD protein (encoded by the *Peptidyl-prolyl cis-trans isomerase F* gene (*Ppif*)) is located in the matrix of the mitochondria, acting as a key regulator in the opening of the mitochondrial permeability transition pore (mPTP) [8,9]. mPTP is a mitochondrial calcium efflux channel and its persistent opening results in deregulated release of calcium and pro-apoptotic factors (i.e. cytochrome c) from the mitochondria triggering the activation of caspases and leading to apoptotic cell death [7,10]. As apoptosis plays a vital part in liver regeneration after two-stage hepatectomies [11], we hypothesized that the modification of mPTP opening could have a considerable effect on the induced hepatic regeneration. An additional consequence of the non-regulated opening of mPTP is the nonspecific transport of aqueous solutes up to a molecular weight of 1500 Da, which results in the depolarization of mitochondria and consequently to the failure of the oxidative phosphorylation [9]. It was already demonstrated that the inhibition of CypD with cyclosporine derivatives could reduce hepatic ischemic injury [12,13], moreover it is important in ischemic-preconditioning [14]. Clinical evidence was also obtained that CypD inhibition could reduce liver cell damage following hepatitis-C infection [15], and quarter-size liver transplantation [16]. These investigations advocate that CypD inhibition could be a therapeutic approach in cell-death-associated diseases. However, to our knowledge there is no literature data on the effect of CypD inhibition following ALPPS. Therefore, we used a CypD knockout (*Ppif -/-*) mice model to investigate how it affects liver regeneration and mitochondrial function following ALPPS.

## 2. Materials and methods

All experiments were reported in accordance with the ARRIVE criteria and were approved by the Scientific Ethical Committee on Animal Experimentation of the National Department of Food Chain Safety (approval number: PEI/001/1732-6/2015). Mice heterozygous for the gene encoding CypD (originating from the C57Bl6/J strain) were obtained from the Dana-Faber Cancer Institute. CypD -/- knockout mice were achieved by mating with C57Bl6/F mice in our facilities and afterwards backcrossing with C57Bl6/J mice for at least eight generations to ensure homologous genetic background. The animals were provided ad libitum access to standard chow (Toxicoop, Hungary) with a temperature (20–22°C) and humidity (40–70%) controlled environment and were kept on a 12-hour day-night cycle.

### 2.1. Operative procedure

Male wild type (WT) BL6/jk (n = 30) and CypD knockout (KO) (n = 30) mice weighing 22–26 g underwent ALPPS procedure. Following 2.5 v/v% with 1 L/min flow isoflurane anaesthesia operation was performed as previously described [5]. Briefly, portal branches leading to the the right lateral, left part of the median, left lateral, and caudate lobes were ligated. Next, alongside the transition line of the median lobe transection was performed with profound electrocauterization of the liver wounds.

### 2.2. Sample extraction

Following 2.5 v/v% with 1 L/min flow isoflurane anaesthesia animals were exsanguinated by cardiopuncture at 24h, 48h, 72h, 168h after surgery. The preoperative group was sacrificed without operation at baseline (N = 6 per group). Body weight was measured before termination (model SCL-1053, Kent Scientific, Torrington, CT, USA).

Tissue from the non-ligated right median lobe (RML) was snap frozen in liquid nitrogen and stored at -80°C until further use or fixed in 4% buffered formaldehyde for histology.

For the isolation of mitochondria from fresh RML samples discontinuous Percoll gradient was used as described earlier [17]. Samples were homogenized in isolation buffer A (S1 Table), then centrifuged (3 min x 1300g). Supernatant was removed and centrifuged again (10 min x 20000g) then pellet was suspended in 15% Percoll and layered on a discontinuous gradient consisting of 40 and 23% Percoll layers, which was then centrifuged (8 min x 30700g). The lowermost fraction of isolation buffer was resuspended, centrifuged (10 x 16,600g), and the pellet was resuspended and centrifuged again (10 x 6300g). Afterwards the supernatant was discharged, then the pellet was resuspended in isolation buffer B (S1 Table).

The retrieved pellet containing isolated mitochondria was resuspended in 200 μl isolation medium (S1 Table). Mitochondrial protein content was determined using Pierce™ Coomassie Plus (Bradford) Assay Kit (Thermo Scientific, Waltham, MA). 0.1 mg/ml mitochondrial protein concentration was used throughout the experiments.

### 2.3. Assessment of mitochondrial function

As mitochondrial function is characterized by oxidative phosphorylation and the intactness of the respiratory chain, adenosine 5′-triphosphate (ATP) synthesis and oxygen consumption was assessed.

#### 2.3.1. Mitochondrial ATP production

Mitochondrial ATP synthesis was investigated as previously described [6]. Incubation medium was supplemented with NADP+ (1.5 mM), hexokinase (2 U/ml), glucose 6-phosphate dehydrogen- ase (3.84 U/ml), 2.5 mM glucose, and 50 μM P1,P5-di(adenosine-5’) pentaphosphate (inhibitor of adenylate kinase). Following coupled enzyme reaction, the absorbance of the reduced nicotinamide adenine dinucleotide phosphate was measured at 340nm (V650 UV/VIS double-beam spectrophotometer, ABL&E Jasco, Tokyo, Japan). ATP production indicating the endogenous substrate supply was assessed in the presence of mitochondria and 2 mM ADP. Stimulated ATP production was evaluated in 5 mM glutamate-malate (GM) (in case of complex I) or 5 mM succinate (in case of complex II) medium.

#### 2.3.2. Mitochondrial oxygen consumption

As previously reported [5,6], oxygen consumption was assessed with an Oxygraph-2K® high resolution respirometry system (Oroboros Instruments, Innsbruck, Austria) by measuring reduced nicotinamide adenine dinucleotide dehydrogenase (I complex) and succinate dehydrogenase (II complex). Basal function (state 4) and induced function stimulated by adenosine 5′-diphosphate (ADP) were measured in GM (complex I) and succinate medium (complex II). Oxygen consumption was adjusted to mitochondrial protein content.

### 2.4. Assessment of intramitochondrial NADP(H)

Intramitochondrial NADP(H) was measured by the autofluorescence of reduced nicotinamide adenine dinucleotide (phosphate) (NAD(P)H) with PTI Deltascan® fluorescence spectrophotometer (Photon Technology International, Lawrenceville, New Jersey, USA) at 37°C, at 344nm excitation and 460nm emission wavelengths. Basal NAD(P)H levels were measured when only mitochondria had been added to the incubation medium. GM or succinate were added for the measurement of induced production [5,6].

### 2.5. Proteomic analysis of mitochondrial biogenesis, oxidative phosphorylation and apoptosis

35 mg of liver tissue was homogenized in RIPA buffer (Sigma-Aldrich, St. Louis, MO) by a Bead Beater tissue homogenizer (Next Advance, Inc, Troy, NY). The electrophoresis of samples (20 μg protein/lane) was performed on 8–12 per cent (v/v) sodium dodecyl sulphate–polyacrylamide gels. Protein concentration was measured using Pierce™ Coomassie Plus (Bradford) Assay Kit (Thermo Scientific, Waltham, MA). After transferring proteins on to polyvinylidene difluoride membranes samples were incubated with primary antibodies (S2 Table). Primary antibodies were detected using secondary antibodies (Jackson ImmunoResearch, West Grove, Pennsylvania, USA) and Clarity ECL reagent (Bio-Rad, Hercules, California, USA). Visualization was carried out on Syngene G:Box imager (Syngene, Cambridge, UK) and quantified with FIJI software [18]. Total protein load of the lane served as internal control. For images of western blots see S1 File.

### 2.6. Quantification of liver mass increase

Liver lobes were weighed separately by an analytical scale (AG245, Mettler Toledo, Greifensee, Switzerland). The following formula was used to calculate increase in liver mass of the non-ligated lobes: (lobe weight/bodyweight at time of death) / (mean lobe weight at preoperative time point/bodyweight at preoperative time point) × 100 (%).

### 2.7. Histology

Tissue samples were embedded in paraffin after they were fixated in 4% paraformaldehyde for 24h, from which liver tissue specimens were made (4-micron-thick histological sections). Specimens were deparaffinized in xylol baths (2x10 min) and rehydrated in graded alcohol series stained with hematoxilin-eosine. Mitotic rate was defined by counting clearly identifiable cromatine divisions/mm2. Ki67 index was assessed as previously described [6]. Antigen was retrieved at pH = 6.0 (S2031, Agilent, Santa Clara, CA,USA). Anti-Ki67 antibodies (ab15580, Abcam, Cambridge, UK) were used to perform Ki67 immunohistochemistry, afterwise with hematoxylin counterstaining was performed. The histological slides were scanned with a Pannoramic P1000 slide scanner system (3DHistech, Budapest, Hungary). Analyzation was performed with QuPath software [19]. The Ki67 index was calculated on the whole slide as the number of Ki67-positive cells per total number of cells [6].

### 2.8. Statistical analysis

Results are presented in mean (s.d.). Normality and homoscedasticity of the data was analyzed with diagnostic tests. Statistical analysis was performed in GraphPad Prism (GraphPad Software, La Jolla, CA, USA). Data was analyzed with a two-way ANOVA with Tukey’s test for post-hoc analysis. A P-value of < 0.05 was considered statistically significant.

## 3. Results

### 3.1. Depletion of Cyclophilin D improves mitochondrial function following ALPPS

Although mitochondrial function showed a significant decrease in the WT group following ALPPS in the first 24–48 h, which is in accordance with our previous results [5,6], it remained preserved overall in the CypD KO group during this time interval. Mitochondrial function was investigated using the parameters described below.

#### 3.1.1. Preserved ATP production in the CypD KO group

**Fig 1:**
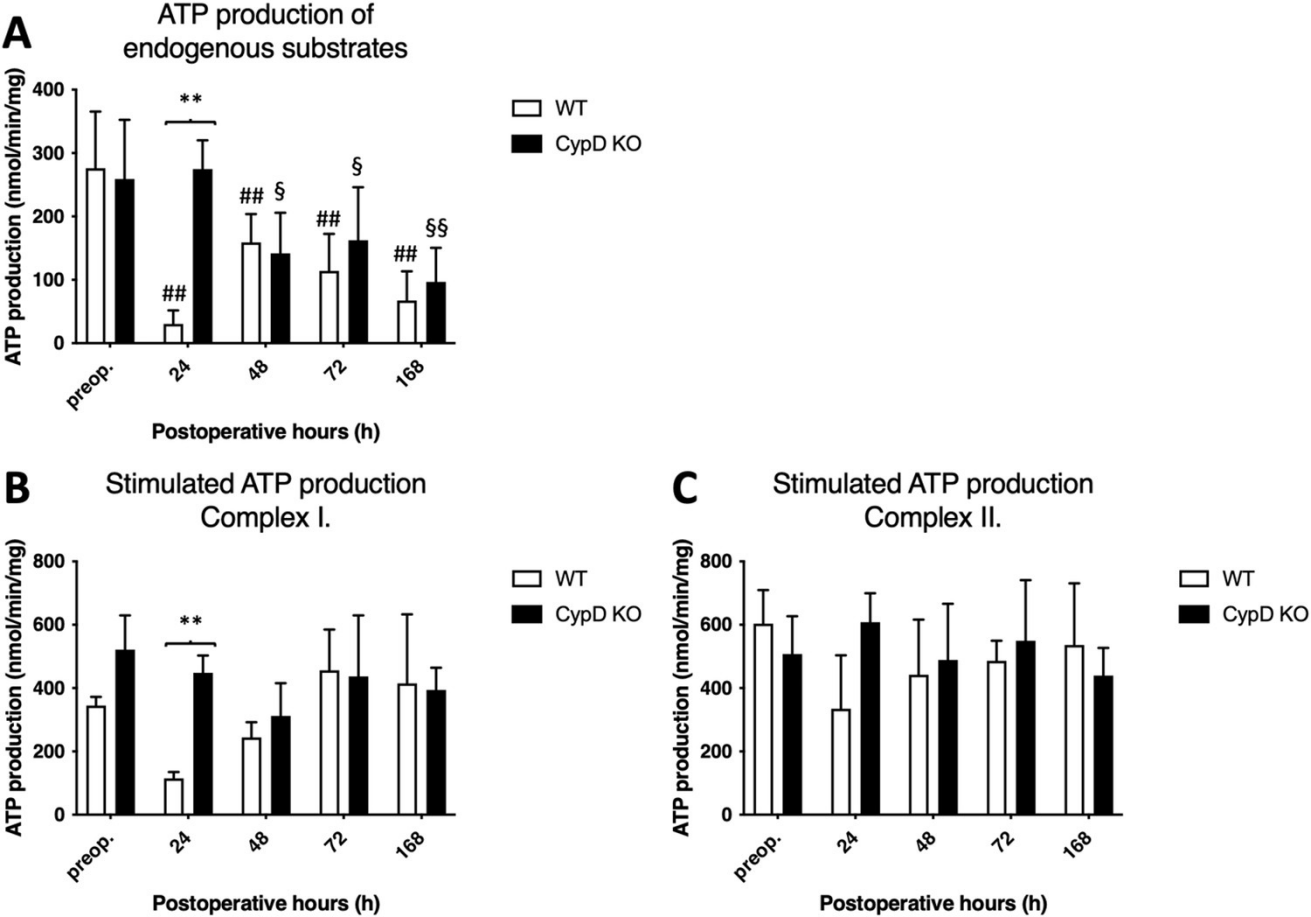
Alterations of adenosine 5′ -triphosphate (ATP) production. Endogenous (A) and exogenous substrate (complex I: Glutamate-malate complex II: Succinate) stimulated (B and C) ATP production preoperatively (preop., 0 h), and at 24 h, 48 h, 72 h, and 168 h after Associating Liver Partition and Portal vein ligation for Staged hepatectomy (ALPPS) (N = 6 per time point per group). * P < 0.050, ** P < 0.0010 versus wild type (WT); # P < 0.050, ## P < 0.001 WT versus corresponding controls (preop.); § P < 0.050, §§ P < 0.0010 CypD KO versus corresponding controls (preop.). Statistical analysis was performed with a two-way ANOVA and Tukey’s post hoc test.

As presented in Fig 1A and 1B, ALPPS resulted in a decrease of ATP production of endogenous substrates from 24 h until 168 h in the WT group, while it was preserved in the CypD KO group at 24 hour and decreased only after from 48 h to 168 h, marking a significant difference between the groups. Stimulated ATP production of complex I showed conjointly a drop at 24 h in the WT group in opposition with preserved value s in the CypD KO group (Fig 1C and 1D).

#### 3.1.2. Enhanced oxygen consumption in the CypD KO group

**Fig 2:**
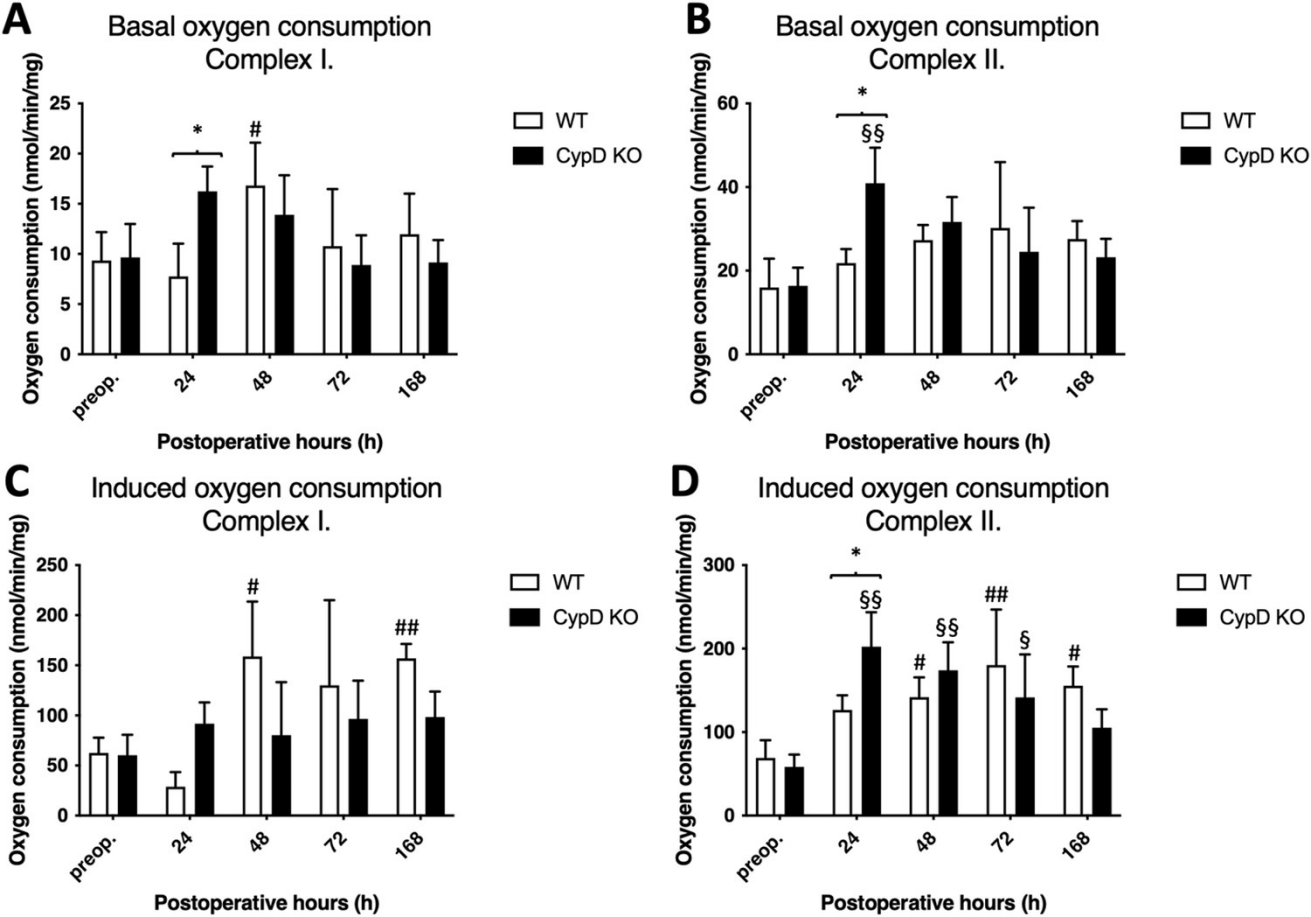
Changes in oxygen consumption levels. Basal (A and B) and substrate (complex I: Glutamate-malate complex II: Succinate) induced (C and D) oxygen consumption preoperatively (preop., 0 h), and at 24 h, 48 h, 72 h, and 168 h after Associating Liver Partition and Portal vein ligation for Staged hepatectomy (ALPPS) (N = 6 per time point per group). * P < 0.050, ** P < 0.0010 versus WT; # P < 0.050, ## P < 0.001 wild type (WT) versus corresponding controls (preop.); § P < 0.050, §§ P < 0.0010 CypD KO versus corresponding controls (preop.). Statistical analysis was performed with a two-way ANOVA and Tukey’s post hoc test.

Basal oxygen consumption remained similar to the preoperative value in the WT group, with a temporary increase of complex I at 48 h. In contrast, CypD KO group peaked at 24 h gradually normalizing by the end of the experiment (Fig 2A and 2B). This resulted in a notably higher oxygen consumption at 24 h in the CypD KO group compared to the WT group. Induced oxygen consumption increased in complex I at 48 and 168 h, while in complex II from 48 h to 168 h in the WT group.

In the CypD KO group complex II showed an earlier increase from 24 h to 72 h, which is line with the energy demanding processes following ALPPS [5]. This resulted in a significantly increased induced oxygen consumption in the CypD KO group compared to the WT group at 24–48 h, in the most energy-consuming phase of liver regeneration following ALPPS (29).

### 3.2. CypD depletion did not alter mitochondrial NAD(P)H content

**Fig 3:**
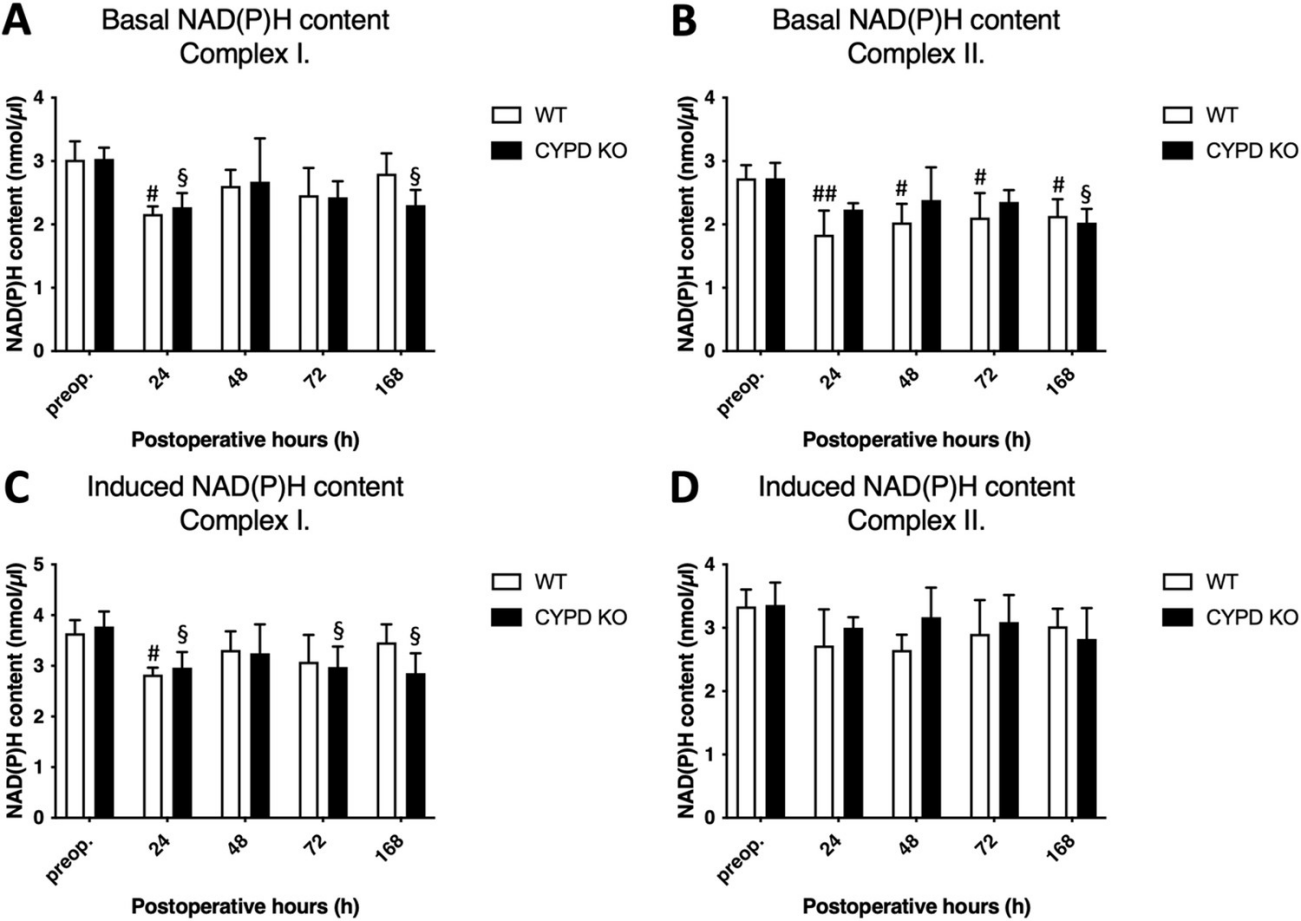
Changes in reduced nicotinamide adenine dinucleotide (phosphate) (NAD(P)H) content. Basal (A and B) and substrate (complex I: Glutamate-malate complex II: Succinate) induced (C and D) NAD(P)H preoperatively (preop., 0 h), and at 24 h, 48 h, 72 h, and 168 h after Associating Liver Partition and Portal vein ligation for Staged hepatectomy (ALPPS) (N = 6 per time point per group). * P < 0.050, ** P < 0.0010 versus wild type (WT); # P < 0.050, ## P < 0.001 WT versus corresponding controls (preop.); § P < 0.050, §§ P < 0.0010 CypD KO versus corresponding controls (preop.). Statistical analysis was performed with a two-way ANOVA and Tukey’s post hoc test.

Our previous results revealed that following ALPPS NAD(P)H content decreases [5]. In line with this, basal concentration of NAD(P)H in the WT group decreased at 24 h in complex I and from 24 h to 168 h in complex II. Corresponding changes could be observed in the CypD KO group, as basal concentration decreased at 24 h and 168 h in complex I and at 168 h in complex II. Therefore, there was no significant difference between the two groups regarding basal NAD(P)H concentration (Fig 3A and 3B). Induced NAD(P)H concentration changed similarly to the basal concentration. A decrease could be observed in the WT group at 24 h in complex I, with no change in complex II. Induced concentration also decreased in the CypD group 24, 72, and 168 h following surgery in complex I, while remained unchanged in complex II. Likewise, there was no difference between the induced NAD(P)H concentration of the two groups (Fig 3C and 3D).

### 3.3. CypD depletion increases mitochondrial biogenesis coordinator PGC1-α level

**Fig 4:**
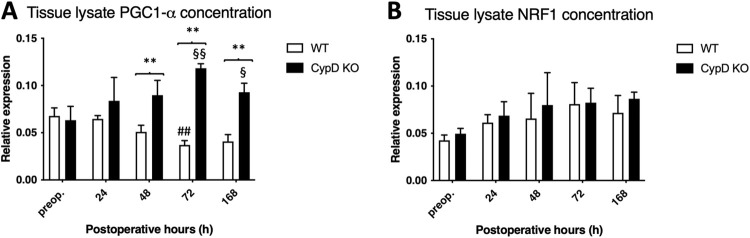
Expression of gene regulatory proteins participating in mitochodrial biogenesis. Peroxisome proliferator-activated receptor γ coactivator (PGC) 1-α (A), nuclear respiratory factor 1 (B) preoperatively (preop., 0 h), and at 24 h, 48 h, 72 h, and 168 h after Associating Liver Partition and Portal vein ligation for Staged hepatectomy (ALPPS) (N = 6 per time point per group). * P < 0.050, ** P < 0.0010 versus wild type (WT); # P < 0.050, ## P < 0.001 WT versus corresponding controls (preop.); § P < 0.050, §§ P < 0.0010 CypD KO versus corresponding controls (preop.). Statistical analysis was performed with a two-way ANOVA and Tukey’s post hoc test.

ALPPS caused the decrease of the mitochondrial biogenesis coordinator PGC1-α at 72 and 168 h in the WT group, which is in accordance with our previous results [5]. On the other hand, CypD depletion increased the level of PGC1-α, as it was found elevated in the CypD KO group from 48h until the end of the experiment. This resulted in a notable difference between the groups from 48 h to 168 h (Fig 4A). Despite of the changes in PGC1-α, NRF 1 level did not change significantly neither in the WT, nor in the CypD KO group (Fig 4B).

### 3.4. CypD depletion increases the production of oxidative phosphorylation system (OXPHOS) proteins

**Fig 5:**
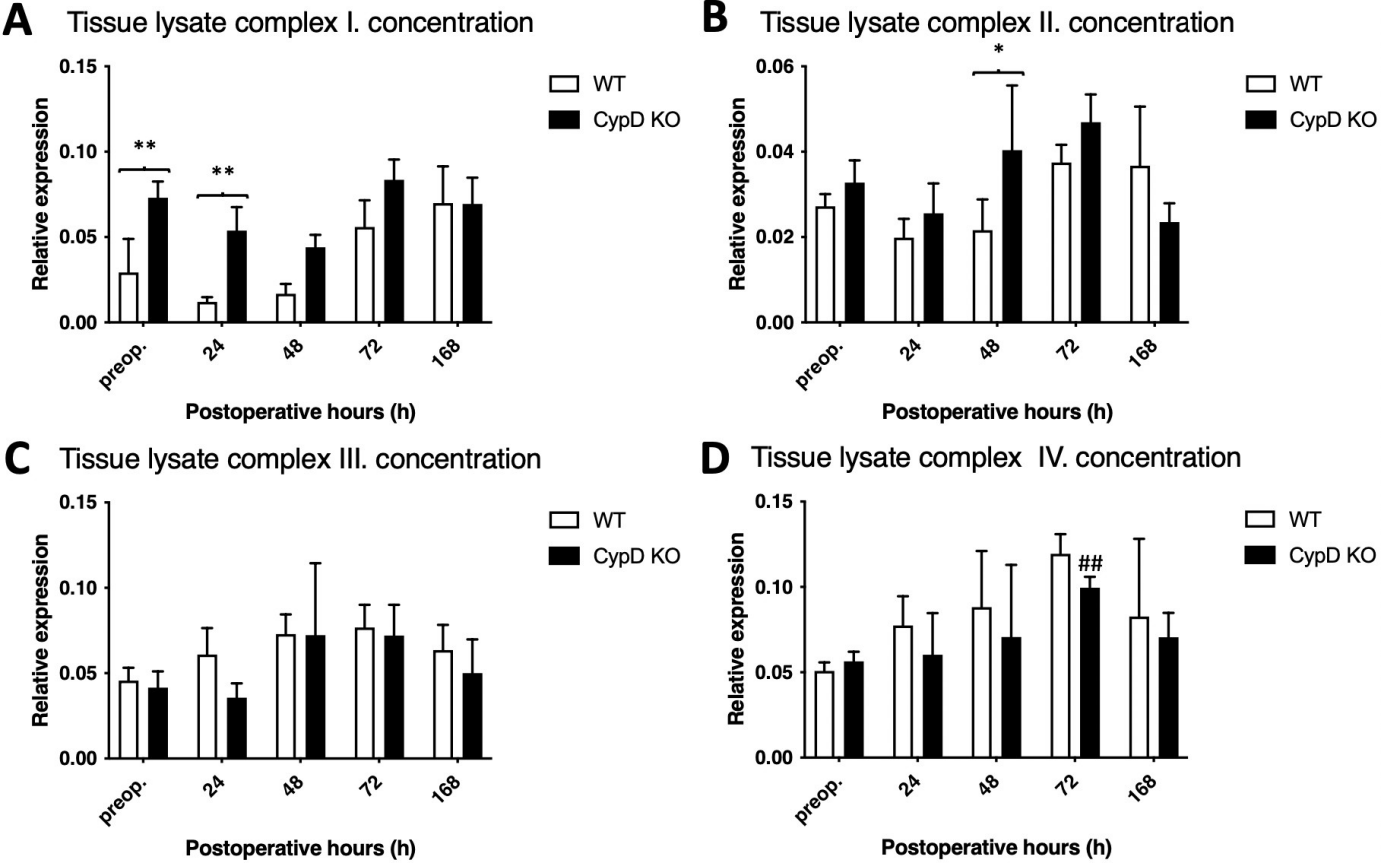
Changes in the expression of the respiratory chain complexes. Complex I (A), complex II (B), complex III (C), complex IV (D) total cell lysate protein concentrations preoperatively (preop., 0 h), and at 24 h, 48 h, 72 h, and 168 h after Associating Liver Partition and Portal vein ligation for Staged hepatectomy (ALPPS) (N = 6 per time point per group). * P < 0.050, ** P < 0.0010 versus wild type (WT); # P < 0.050, ## P < 0.001 WT versus corresponding controls (preop.); § P < 0.050, §§ P < 0.0010 CypD KO versus corresponding controls (preop.). Statistical analysis was performed with a two-way ANOVA and Tukey’s post hoc test.

In the WT group OXPHOS complex I-III levels did not change significantly throughout the experiment, while in case of complex IV a temporary increase could be observed at 72 h. Similarly, OXPHOS complex I-IV levels in the CypD KO group did not change during the experiment. However, due to an initially higher level at the baseline, level of OXPHOS complex I the CypD KO group was higher compared to the WT group preoperatively and at 24 h, in case of complex II the same disparity could be observed at 48 h, at the most vulnerable time points following ALPPS (Fig 5A–5D).

### 3.5. Altered activation of apoptotic caspase-3 in the CypD KO group

**Fig 6:**
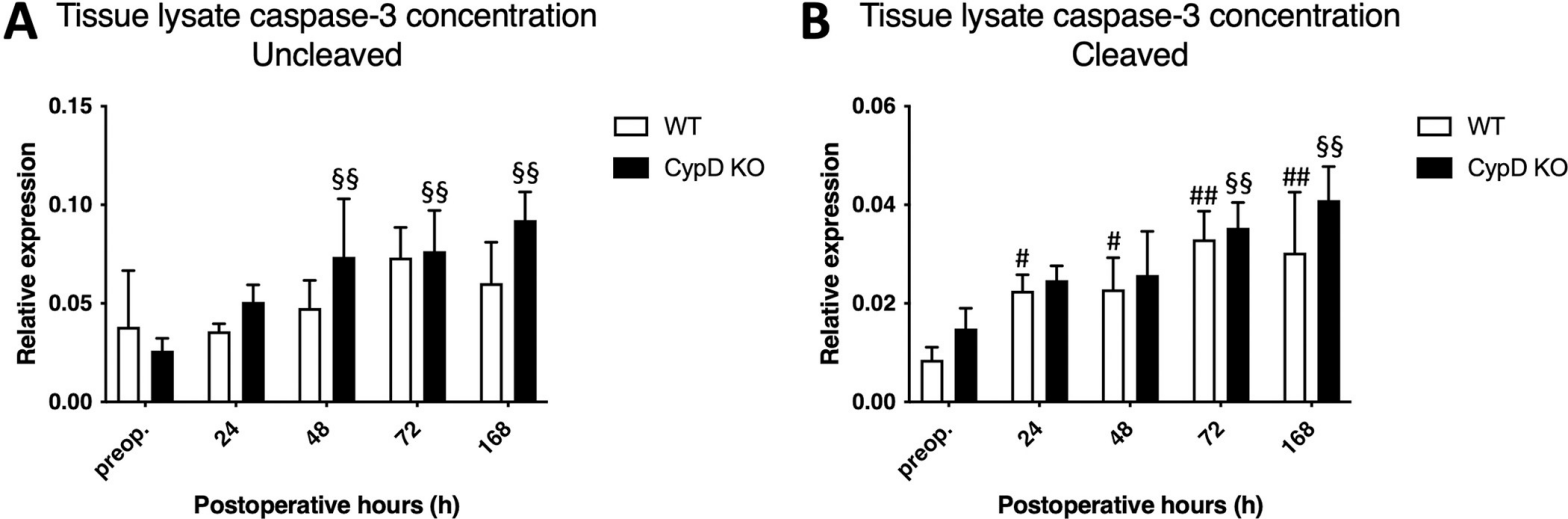
Expression of caspase-3. Uncleaved (A) and cleaved (B) caspase-3 total cell lysate protein concentrations preoperatively (preop., 0 h), and at 24 h, 48 h, 72 h, and 168 h after Associating Liver Partition and Portal vein ligation for Staged hepatectomy (ALPPS) (N = 6 per time point per group). * P < 0.050, ** P < 0.0010 versus wild type (WT); # P < 0.050, ## P < 0.001 WT versus corresponding controls (preop.); § P < 0.050, §§ P < 0.0010 CypD KO versus corresponding controls (preop.). Statistical analysis was performed with a two-way ANOVA and Tukey’s post hoc test.

The level of uncleaved proenzyme form of caspase-3 did not change in the WT group, while a significant increase could be observed from 48 h until the end of the experiment in the CypD KO group (Fig 6A). The level of the activated cleaved form of caspase-3 was elevated at 24 h in the WT group and remained increased by the end of the experiment, which is in line with previous results on apoptotic activity following ALPPS. In contrast, active caspase-3 level in the CypD KO group increased significantly only from 72 h to168 h, in the remodeling period of ALPPS (Fig 6B).

### 3.6. CypD depletion accelerates liver growth and cell proliferation

**Fig 7:**
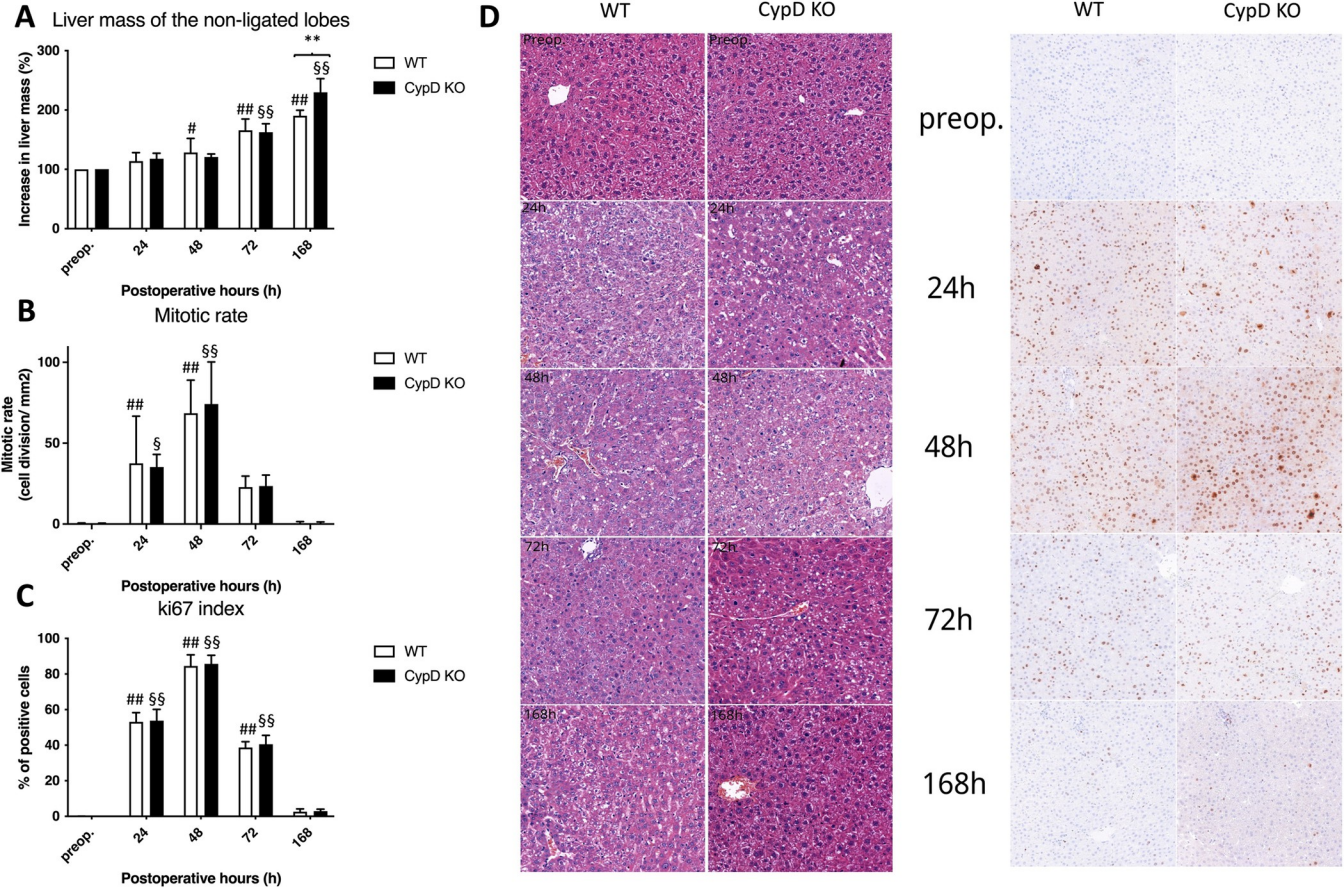
Liver regeneration following Associating Liver Partition and Portal vein ligation for Staged hepatectomy (ALPPS) in the ligated right medial lobe. Increase in liver mass of non-ligated lobe (A) and mitotic rate (B) and ki67 index (C) preoperatively (preop., 0 h), and at 24 h, 48 h, 72 h, and 168 h after ALPPS (N = 6 per time point per group). Histological structure on the left (hematoxylin and eosin stain; original magnification: ×150) and ki67 immunohistochemistry (on the right) of the regenerating right median (RM) lobe (D) preoperatively (preop.) and at 24 h, 48 h, 72 h, and 168 h after ALPPS in the Cyclopihilin D knockout (CypD KO) and wild type (WT) group. * P < 0.050, ** P < 0.0010 versus wild type (WT); # P < 0.050, ## P < 0.001 WT versus corresponding controls (preop.); § P < 0.050, §§ P < 0.0010 CypD KO versus corresponding controls (preop.). Statistical analysis was performed with a two-way ANOVA and Tukey’s post hoc test.

Both in the WT and CypD KO groups, liver mass gradually increased. However, while liver growth achieved only about 100% growth in the WT group, the CypD KO group displayed a 150% growth, resulting significantly higher liver mass in the CypD KO group at 168 h compared to the WT group (Fig 7A). Mitotic rate and ki67 index in both WT and CypD KO group increased until 48 h, after which the cell division gradually normalized by the end of the experiment. No difference could be observed nor between the mitotic rates either the ki67 index of the groups (Fig 7B–7D).

## 4. Discussion

ALPPS is a novel modification of the classic two-stage hepatectomy, allowing for the extended resection of liver tumors considered inoperable by standard procedures. By propagating a robust and rapid regeneration ALPPS can eliminate the disadvantages of classic portal vein occlusional techniques [2]. However, the initially high morbidity and mortality rates experienced after the operation raised skepticism [2]. These phenomena could be explained by the delayed functional recovery of hepatocytes appertaining to the non-ligated regenerating lobes [4]. As previously shown in our study, a key component of this might be the energetic disbalance caused by the insufficiency of mitochondria [5]. Therefore, we hypothesized that the disadvantageous aspects of ALPPS could be countered with mitochondrial therapy. As a first step to verify our hypothesis, our aim was to explore possible therapeutic approaches to boost mitochondrial function following ALPPS. Although in our previous study we found that preoperative exercise could greatly enhance mitochondrial function and substantiate even more powerful regeneration [6], we aimed to seek for pharmacological therapeutic options to complement and support physical prehabilitation. As CypD inhibition has been previously proven an effective mitochondrial therapy in a variety of pathophysiological conditions [7], here we investigated the effect of CypD depletion on mitochondrial function, biogenesis and liver regeneration in CypD KO mice undergoing ALPPS.

With this approach we found in the CypD KO mice (a) improved mitochondrial function; (b) an elevated level of mitochondrial biogenesis coordinator PGC1-α and increased production of OXPHOS proteins; (c) a delay in the activation of caspase-3, which resulted in apoptotic activity only in the late, remodeling period of ALPPS-induced liver regeneration; and (d) accelerated liver growth.

ALPPS is associated with increased morbidity and mortality rate along with a delayed functional recovery of the regenerating liver [3,4]. Following hepatectomy, an increased energy supply is necessary for cell division [10]. Considering that ALPPS forces even more hepatocytes into proliferation [2], there is no doubt that it is a highly energy-dependent process. However, our previous study showed impaired mitochondrial function and biogenesis following ALLPS [5], which together with the increased energy demand could be the underlying reason behind the unfavorable morbidity and mortality rates. Supporting our previous data, mitochondria, isolated from WT mice exhibited decreased oxygen consumption and decreased ATP production demonstrating the impairment of mitochondria after ALLPS. On the other hand, mitochondrial functions were better preserved in the CypD KO mice as indicated by the increased ATP production and oxygen consumption. Our results are supported by the putative framework about the effects of CypD inhibition on mitochondrial function and cell energy supply [9,16,20]. As CypD facilitates the opening of mPTP, it contributes to a striking increase in the permeability of the inner membrane, dissipation of the mitochondrial membrane potential and interruption of ATP production [21]. Therefore, as also shown by our results, inhibition of CypD could indeed serve as a pharmacological target to improve mitochondrial function following ALPPS.

In order to explore the wider effects of CypD depletion on the process of energy production, we have also investigated the intramitochondrial NAD(P)H content. Our previous study showed a decrease in NAD(P)H steady state following ALPPS [5]. According to this, NAD(P)H concentration decreased in WT mice following surgery. A similar decrease could also be observed in CypD KO mice, which suggests that NAD(P)H content was not influenced, only the process of the terminal oxidation. However, considering that during liver proliferation the primary energy source of hepatocytes is oxidative phosphorylation [10], which was improved in the CypD KO group, CypD depletion seems beneficial on the cell energy supply.

ALPPS also deteriorates mitochondrial biogenesis and protein production by a prolonged and overwhelming inflammatory response. In the course of this, tumor necrosis factor (TNF)-alpha reduces the level of PGC-1alpha directly by nuclear factor kappa-light-chain-enhancer of activated B cells (NF-κB) resulting in mitochondrial dysfunction [5,6]. It has been also shown that CypD depletion attenuates inflammatory response by reducing TNF-alpha and NF-κB [22]. Therefore, we hypothesized that CypD depletion has an impact on the PGC1-α mitochondrial biogenesis pathway and the transcription of mitochondrial proteins following the operation. In accordance with our previous results [5], PGC1-α level was strongly supressed in the WT group after ALPPS, indicating damaged mitochondrial biogenesis. By contrary, the CypD KO group displayed an increased level of PGC1-α compared both to the WT group and the preoperative state. However, the increase of PGC1-α was not followed by NRF1, which is a major transcriptional factor of mitochondrial respiratory complexes [23]. Therefore, it was also a compelling question whether the protein complexes of the OXPHOS system were affected by CypD depletion. While numerous studies demonstrate that CypD appears to regulate the OXPHOS system by various kinds of mechanisms [24], currently to our knowledge there is no literature data about the transcriptional effects of CypD inhibition on the OXPHOS system. Our results showed increased protein levels in case of complex I and II in the CypD KO group while no change could be detected in case of complex III-IV.

The opening of the mPTP, controlled by CypD, could lead to cell death in two major ways. First, failing of the oxidative phosphorylation via the depolarization of the mitochondrial membrane leads to necrosis [25]. Second, the opening causes the swelling of the mitochondria contributing to the rupture of the outer membrane and the release of cytochrome c from the inner space, which activates caspases and apoptosis through the inhibition of PARP activity [7,26]. As we have demonstrated that CypD depletion improves oxidative phosphorylation, we also aimed to investigate how it affects mitochondrial apoptosis. Strikingly, a lagging activation of caspase-3 could be observed in the CypD KO group, which resulted in apoptotic activity only from 72 h to 168 h. In contrast, the WT group displayed an increase in activated caspase-3 level already after 24 h. To understand the importance of this, it is very vital for the reader to contextualize the two important phases of regeneration through which the non-ligated liver lobe undergoes after ALPPS. (1) The initial phase starts right after the surgery and lasts 48 h, when increased portal pressure and microcirculation along with cytokines activate the regeneration cascade, making this phase the most vulnerable one after surgery; (2) the remodelling period takes place from 72 h to 168 h, when apoptotic cell death is necessary for the removal of redundant hepatocytes together with the reconstruction of hepatic cords [11]. Although further investigations are needed, our results suggest that by stabilizing the mitochondria CypD depletion could also influence apoptosis following ALPPS eventuating in the delay of apoptotic activation until the favourable remodelling period instead of the initial, vulnerable phase.

Our last compelling question was whether liver regeneration was affected in the CypD KO group, as literature data points to a correlation between mitochondrial functioning and regeneration. By buffering mitochondrial Ca^2+^, augmented liver regeneration can be achieved via inhibited apoptosis and a direct effect on cell proliferation controlled by the shift in B-cell lymphoma protein 2 (Bcl-2)-associated X (Bax)/Bcl-2 after partial hepatectomy [27]. In an experimental model of quarter-size liver transplantation liver regeneration was stimulated with a CypD inhibitor (NIM811) [16]. Following ALPPS, when already deteriorated mitochondrial function is entailed [5], toxin-induced fibrosis results in an even more explicit mitochondrial dysfunction, which attenuates the rapid regenerative capacity of ALPPS [28]. Adding to this, our previous investigation revealed improved mitochondrial function and strongly induced regeneration after ALPPS as an effect of preoperative exercise [6]. Considering the above, we hypothesized that CypD depletion could also have a positive effect on liver regeneration following ALPPS. In line with the above, our results demonstrated increased liver growth in the CypD KO group compared to the WT mice. However, in the extent of cell division no substantive difference could be observed. The discrepancy between the liver growth and the cell division could be explained by a decreased apoptosis in the CypD KO group.

The limitations of the study must be acknowledged, as physiological differences between rodents and humans might affect this model. However, energy production, mitochondrial structure and biogenesis are extremely conserved in mammals [29], as well as the PPIF gene encoding CypD in vertebrates [30], therefore data gained from rodents could be appropriately translated to humans.

In conclusion, our study proved that mitochondrial function could be stabilized by the depletion of CypD, which could contribute to the mitigation of the vulnerability following ALPPS. To our best knowledge, the effect of mitochondrial treatment has not yet been investigated in rapport with ALPPS. Although further analysis regarding the pharmacological inhibition of CypD are essential, so far our previous [5,6] and present results underline the relevance of mitochondrial therapy after ALPPS. Therefore, we propose that CypD inhibition should be further investigated as a potential pharmacological target to enhance the post-operative outcomes following ALPPS.

## Supporting information

S1 Table Antibody list. (DOCX)

S2 Table Reaction medium compositions. (DOCX)

S3 Table Data sets. (XLSX)

S1 File Western blotts. (DOCX)
