# Supplementary material for: The effect of Cyclophilin D depletion on liver regeneration following associating liver partition and portal vein ligation for staged hepatectomy
Source: PLoS One. 2022 Jul 14;17(7):e0271606. doi: 10.1371/journal.pone.0271606 (PMC9282546; doi:10.1371/journal.pone.0271606)
Supplement: S1 Table — (DOCX) [file pone.0271606.s001.docx]

S1 Table: Reaction medium compositions

| **Medium** | **Components (mM)** | **pH** |
| --- | --- | --- |
| **Mitochondria isolation medium A** | 225 mannitol, 75 sucrose, 5 Hepes, 1 EGTA, | pH 7.4 (KOH) |
| **Mitochondria isolation medium B** | 225 mannitol, 75 sucrose, 5 Hepes, | pH 7.4 (KOH) |
| **Incubation medium for O_2_ consumption, ATP production, NAD(P)H concentration measurements** | 125 KCl, 20 Hepes, 2 K2HPO4, 1 MgCl2, 0.1 EGTA, supplemented with 0.025% fatty-acid- free bovine serum albumin | pH 7.0  (KOH) |

O_2_: Oxygen, ATP: Adenozin triphosphate, NAD(P)H: Nicotinamide adenine dinucleotide (phosphate) ROS: Reactove Oxygen Species, KCL: Potassium Cloride, K_2_HPO_4_: Dipotassium phosphate, MgCl_2_: Magnesium Cloride, EGTA: egtazic acid, BSA: Bovine serum albumin
