# Supplementary material for: The effect of Cyclophilin D depletion on liver regeneration following associating liver partition and portal vein ligation for staged hepatectomy
Source: PLoS One. 2022 Jul 14;17(7):e0271606. doi: 10.1371/journal.pone.0271606 (PMC9282546; doi:10.1371/journal.pone.0271606)
Supplement: S2 Table — (DOCX) [file pone.0271606.s002.docx]

S2 Table: Antibody list

| Antigen | Catalog number | Manufacturer | Dilution and application | |
| --- | --- | --- | --- | --- |
| PGC1-α | #KP-9803 | Merck (Darmstadt, Germany) | 1/9000 | WB |
| NRF1 | #sc-33771 | Santa Cruz (Santa Cruz, CA, USA) | 1/5000 | WB |
| OXPHOS rodent mix   - CI subunit NDUFB8 - CII-Iron-sulfur protein (IP) subunit of succinate dehydrogenase (SDH) - CIII-Core protein 2 - CIV subunit I | #ab110413  (ab110242)  (ab14714)  (ab14745)  (ab14705) | Abcam (Cambridge, UK) | 1/4000 | WB |
| Caspase-3 | #14220 | Cell Signaling (Danvers, Massachusetts, USA) | 1/1000 | WB |
| Cleaved Caspase-3 | #9664 | Cell Signaling (Danvers, Massachusetts, USA) | 1/1000 | WB |
| Goat anti-mouse (secondary)(HRP) | #R-05071-500 | Advansta (San Jose, Ca USA) | 1/5000 | WB |
| Goat anti-rabbit (secondary)(HRP) | #R-05072-500 | Advansta (San Jose, Ca USA) | 1/5000 | WB |
| Ki67 | ab15580 | Abcam (Cambridge, UK) | 1/100 | IC |
| Rabbit IgG VisUCyte HRP Polymer Antibody | VC002 | R & D Systems  (Minnesota, USA) | RTU | IC |

WB: western blot
IC: immunhistochemistry
RTU: ready to use
