# Supplementary figures and images for: The effect of Cyclophilin D depletion on liver regeneration following associating liver partition and portal vein ligation for staged hepatectomy
Source: PLoS One. 2022 Jul 14;17(7):e0271606. doi: 10.1371/journal.pone.0271606 (PMC9282546; doi:10.1371/journal.pone.0271606)

Supplementary file 1: Western blotts

**
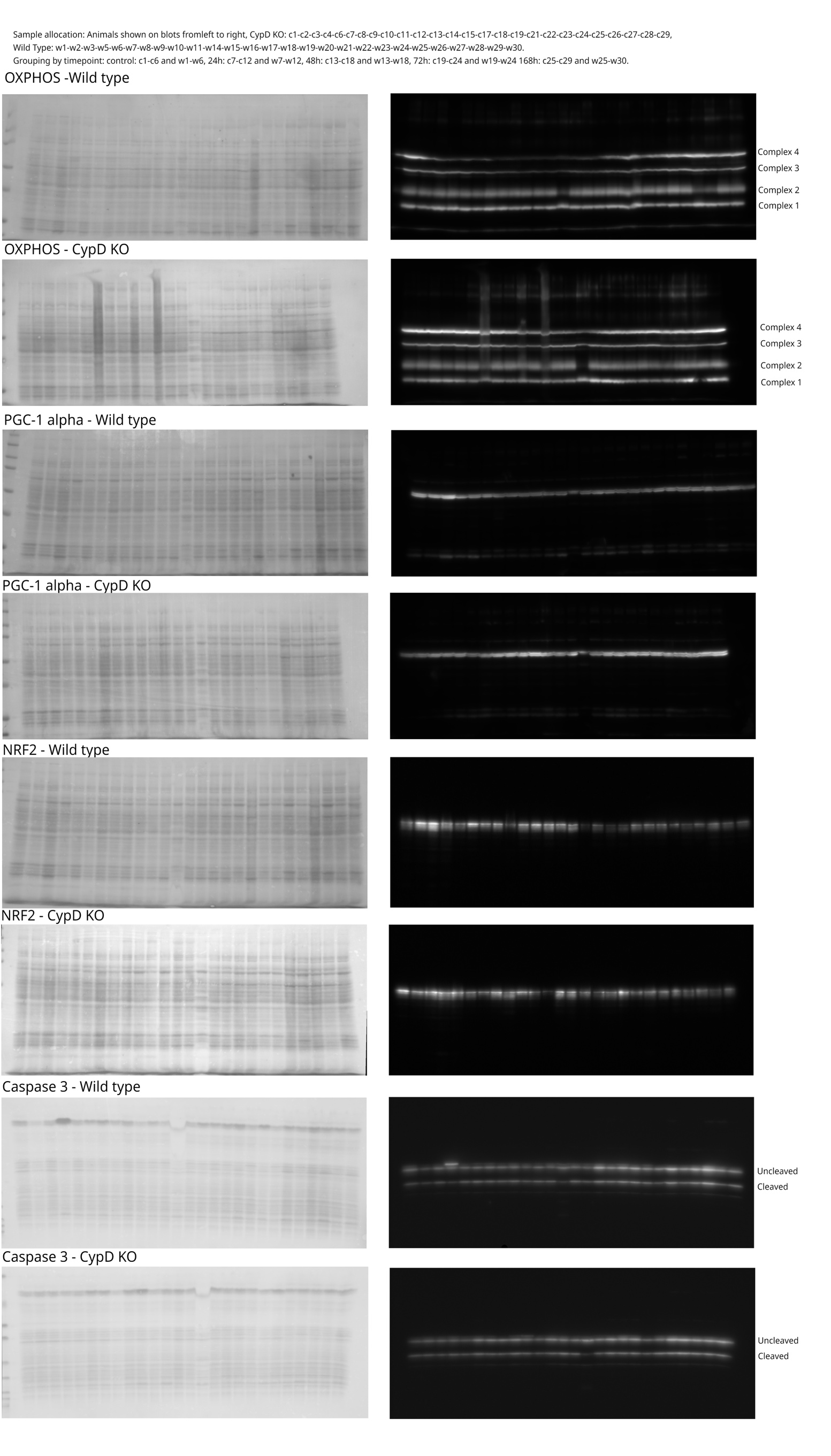
**

**
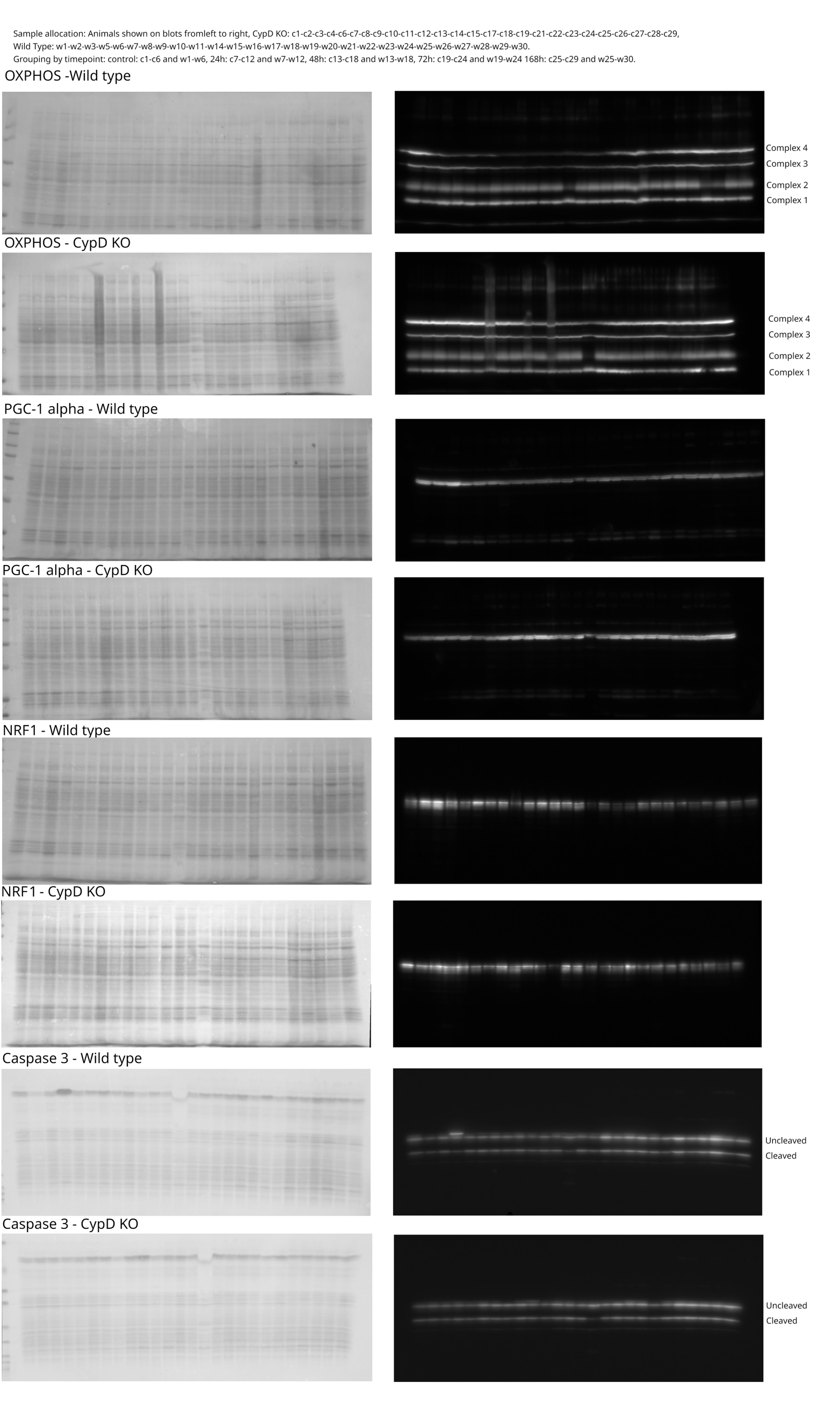
**

Supplement: S1 File — (DOCX) [file pone.0271606.s004.docx]
